# Hypoxia equally reduces the respiratory compensation point and the NIRS‐derived [HHb] breakpoint during a ramp‐incremental test in young active males

**DOI:** 10.14814/phy2.14478

**Published:** 2020-06-26

**Authors:** Rafael D. A. Azevedo, Béjar Saona J. E., Erin Calaine Inglis, Danilo Iannetta, Juan M. Murias

**Affiliations:** ^1^ Faculty of Kinesiology University of Calgary Calgary AB Canada

**Keywords:** cycling, deoxygenation breakpoint, exercise intensity, RCP, thresholds

## Abstract

This study investigated the effect of reduced inspired fraction of O_2_ (FiO_2_) in the correspondence between the respiratory compensation point (RCP) and the breakpoint in the near‐infrared spectroscopy‐derived deoxygenated hemoglobin signal ([HHb]_bp_) during a ramp‐incremental (RI) test to exhaustion. Eleven young males performed, on two separated occasions, a RI test either in normoxia (NORM, FiO_2_ = 20.9%) or hypoxia (HYPO, FiO_2_ = 16%). Oxygen uptake ( $\dot{V}$O_2_), and [HHb] signal from the vastus lateralis muscle were continuously measured. Peak $\dot{V}$O_2_ (2.98 ± 0.36 vs. 3.39 ± 0.26 L min^−1^) and PO (282 ± 29 vs. 310 ± 19 W) were lower in HYPO compared to NORM condition, respectively. The $\dot{V}$O_2_ and PO associated with RCP and [HHb]_bp_ were lower in HYPO (2.35 ± 0.24 and 2.34 ± 0.26 L min^−1^; 198 ± 37 and 197 ± 30 W, respectively) when compared to NORM (2.75 ± 0.26 and 2.75 ± 0.28 L min^−1^; 244 ± 29 and 241 ± 28 W, respectively) (*p* < .05). Within the same condition, the $\dot{V}$O_2_ and PO associated with RCP and [HHb]_bp_ were not different (*p* > .05). Bland–Altman plots mean average errors between RCP and [HHb]_bp_ were not different from zero in HYPO (0.01 L min^−1^ and 1.1 W) and NORM (0.00 L min^−1^ and 3.6 W) conditions. The intra‐individual changes between thresholds associated with $\dot{V}$O_2_ and PO in HYPO from NORM were strongly correlated (*r* = .626 and 0.752, *p* < .05). Therefore, breathing a lower FiO_2_ during a RI test resulted in proportional reduction in the RCP and the [HHb]_bp_ in terms of $\dot{V}$O_2_ and PO, which further supports the notion that these physiological responses may arise from similar metabolic changes reflecting a common phenomenon.

## Graphical abstract

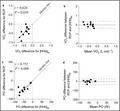

## 1 INTRODUCTION

During a ramp‐incremental (RI) test to exhaustion, the rate of O_2_ uptake ( $\dot{V}$O_2_) increases rather linearly from the exercise onset until maximal $\dot{V}$O_2_ ( $\dot{V}$O_2max_) or task failure ensue. At approximately 80% of $\dot{V}$O_2max_, two physiological responses become discernible: at the pulmonary level the respiratory compensation point (RCP) (Whipp, Davis, & Wasserman, 1989) is expressed, whereas at the level of the muscle (i.e. the *vastus lateralis* muscle), a breakpoint in the near‐infrared spectroscopy (NIRS)‐derived deoxygenated hemoglobin signal ([HHb]_bp_) is observed (Spencer, Murias, & Paterson, 2012).

At the pulmonary level, the RCP is identified as the onset of a more rapid increase in minute ventilation that is disproportional from the rate of carbon dioxide production (V̇CO_2_), which causes the arterial tension of CO_2_ to fall (Whipp et al., 1989). This hyperventilatory response offers partial compensation to the blood accumulation of hydrogen ions ([H^+^]) arising from the accelerated glycolytic rate within the active musculature (Whipp et al., 1989). At the muscle level (within the vastus lateralis of the quadriceps muscles), the [HHb]_bp_ demarcates the beginning of a plateau‐like response in the [HHb] signal (Spencer et al., 2012), with the adjustment of the [HHb] being a proxy for local O_2_ extraction, as it represents the balance between delivery and off‐loading of O_2_ to and out of the capillary microvasculature, respectively. (Grassi & Quaresima, 2016). Although still a subject of debate, our group has suggested that the [HHb]_bp_ and the subsequent plateau might be related to improved blood flow redistribution toward the active tissues of the of the vastus lateralis (Azevedo, Béjar Saona, Inglis, Iannetta, & Murias, 2020; Inglis, Iannetta, & Murias, 2017; Murias, Spencer, Keir, & Paterson, 2013), whereby the plateau in the [HHb] signal would result from an increased local O_2_ delivery in the presence of a continuous increase in $\dot{V}$O_2_ (Murias, Spencer, et al., 2013). However, others have proposed that the occurrence of the plateau in the [HHb] signal might be due to other physiological factors, such as the achievement of the upper limit for O_2_ extraction in the superficial portions of muscle (Okushima et al., 2015), and/or limitations to perfusive or diffusive provision of O_2_ during high‐intensity exercise (Okushima et al., 2020).

Representative of whole‐body and local physiological responses, the RCP and the [HHb]_bp_, respectively, have been suggested to indicate the metabolic rate associated with the transition from the heavy into the severe exercise intensity domains (Keir et al., 2015; Keir, Pogliaghi, & Murias, 2019). This suggestion has been based on studies that have shown their correspondence on the basis of $\dot{V}$O_2_ and power output (PO) during RI tests (Fontana et al., 2015; Iannetta, Passfield, Murias, Calaine, & Christopher, 2018; Iannetta, Qahtani, Millet, & Murias, 2017; Inglis, Iannetta, Keir, & Murias, 2019), and their occurrence at a $\dot{V}$O_2_ similar to that associated with the maximal lactate steady state (MLSS) and critical power (CP) (Bellotti, Calabria, Capelli, & Pogliaghi, 2013; Iannetta, Passfield, et al., 2018; Keir et al., 2015). In this context, a recent report has demonstrated that the RCP and the [HHb]_bp_, (as well as MLSS) of trained cyclists changed uniformly during the course of a competitive season (Inglis et al., 2019). Despite these lines of evidence, however, the idea of an equivalence between the RCP and the [HHb]_bp_ is still under debate and other research groups have suggested plausible alternative interpretations (Boone, Barstow, Celie, Prieur, & Bourgois, 2016; Broxterman, Craig, & Richardson, 2018; Caen, Vermeire, Bourgois, & Boone, 2018). Therefore, interventions that can alter the occurrence of both the RCP and the [HHb]_bp_ are needed to further test whether these two indices represent a similar physiological event during RI exercise and, thus, can possibly be seen as equivalent.

From this perspective, breathing hypoxic air is an intervention that can be used to potentially alter the occurrence of both the RCP and [HHb]_bp_ during a RI test, and thus provide insights into the relationship between these two physiological indices of exercise intensity (Leo, Sabapathy, Simmonds, & Cross, 2017). Indeed, hypoxia has been shown to limit O_2_ provision to the peripheral tissues as indicated by a reduction in arterial O_2_ saturation and capillary partial pressure of O_2_ (Richardson et al., 2006). These impairments typically result in lower $\dot{V}$O_2max_ and peak power output (PPO) during incremental exercise (Knight et al., 1992), and a smaller PO associated with the heavy‐to‐severe boundary of exercise intensity, as demonstrated by reduced CP in hypoxia (La Monica et al., 2018; Parker Simpson, Jones, Skiba, Vanhatalo, & Wilkerson, 2014; Townsend, Nichols, Skiba, Racinais, & Périard, 2017). Thus, it could be expected that breathing a lower fraction of O_2_ (FiO_2_) during a RI test would also affect the RCP and the [HHb]_bp_ by reducing the $\dot{V}$O_2_ and PO at which they occur with respect to normoxia. Indeed, previous findings have shown a reduction in the PO associated with the [HHb]_bp_ in hypoxia compared to normoxia (Azevedo et al., 2020; Osawa, Kime, Hamaoka, Katsumura, & Yamamoto, 2011). Although suggested by a previous study (Osawa et al., 2011), no investigation has quantified if the changes in the RCP and the [HHb]_bp_ in hypoxia compared to normoxia would be of similar magnitude, and, thus, be linked to one another.

Therefore, this study explored the changes in the $\dot{V}$O_2_ and PO associated with the RCP and the [HHb]_bp_ in response to a diminished FiO_2_ (16%) during a RI test to exhaustion. It was hypothesized that a reduction in both the $\dot{V}$O_2_ and PO at the RCP and the [HHb]_bp_ would occur as a consequence of the decreased FiO_2_, and that these changes would be proportional on an individual basis.

## 2 METHODS

### 2.1 Participants

Eleven healthy males were recruited (age: 29 ± 6 years; height: 179 ± 6 cm; weight: 79 ± 9 kg). Participants gave informed written consent to participate in this study after completing the physical activity readiness questionnaire (PAR‐Q+) and being cleared for exercise. All participants were free of any medical condition that could have altered their normal cardiovascular responses to exercise. The study was approved by the Conjoint Health Research Ethics Board at the University of Calgary. This study was part of a larger project designed to answer different research questions (Azevedo et al., 2020), whereby, out of 11 participants, 10 were included in our previous publication.

### 2.2 Experimental design

Participants reported to the laboratory on two separate occasions at similar time of the day (±1 hr). The visits were separated by at least 48 hr, during which participants performed a RI test to exhaustion either in normoxia (NORM, FiO_2_ = 20.9%) or hypoxia (HYPO, FiO_2_ = 16%). The FiO_2_ for hypoxia was chosen with reference of previous investigations demonstrating that this specific FiO_2_ was effective in altering arterial and muscle O_2_ saturation during exercise (Amann, Romer, Subudhi, Pegelow, & Dempsey, 2007). Before each RI test, a moderate intensity step transition, consisting of cycling at 20 W for 6 min and at 80 W for another 6 min, was performed to subsequently compute the mean response time (MRT) of $\dot{V}$O_2_ (Iannetta, Murias, & Keir, 2019) (see data analyses for details). Thereafter, the participants were given 2 min of rest followed by a 4 min baseline cycling at 20 W prior to the RI test. The RI tests to volitional exhaustion consisted of 30 W min^−1^ continuous increments (i.e. 1 W every 2 s). Each exercise session was preceded by a 5 min wash‐in period with the predetermined FiO_2_ (either hypoxia or normoxia), during which participants rested seated on the bike. All exercise testing was performed on an electro‐magnetically braked cycle ergometer (Velotron Dynafit Pro; Racer Mate).

### 2.3 Measurements

#### 2.3.1 Pulmonary O_2_ uptake

A breath‐by‐breath metabolic cart system (Quark CPET, Cosmed), which was calibrated before each test as per the manufacturer's recommendations, was used to measure ventilatory and gas exchange variables. The expiratory and inspiratory air volume rates and the concentrations of inspired and expired O_2_ and CO_2_ were measured through a low‐resistance flowmeter and high precision gas analyzers, respectively. During all sessions, participants breathed through a mask connected to the flowmeter which was attached to a two‐way low‐resistance T‐valve (Hans Rudolph In.; 2600 series, medium two‐way NRBV). To control the FiO_2_ during HYPO, inspired air was delivered by a gas‐mixing device (Altitrainer NP, SMTEC) connected with a tube to the T‐valve to provide the desired concentrations of O_2_. During NORM, the same system was used to deliver room air in order to blind participants from the experimental condition.

#### 2.3.2 NIRS‐derived signals

[HHb] (µM) and tissue O_2_ saturation index (StO_2_) (%) signals of the VL muscle were measured using a two‐channel frequency‐domain NIRS device (Oxiplex TS; ISS) at a sampling rate of 2 Hz, as described elsewhere (Iannetta, Qahtani, Millet, et al., 2017). The probe was placed on the belly of VL muscle, midway between the greater trochanter and the proximal border of the patella and secured in place by double‐sided tape as well as an elastic strap to prevent movement. Additionally, the NIRS probe was covered with an optically dense, black vinyl sheet to minimize possible intrusion of extraneous light. Before removing the probe, the area was marked to ensure the consistency of the placement for the following visit. The NIRS system was calibrated before each test, as per the manufacturer's recommendations.

#### 2.3.3 Pulse O_2_ saturation

The pulse oxygen saturation (SpO_2_) was measured via a pulse oximetry pod (ADInstruments) from the forefinger of the right hand at a 1 Hz sampling rate and at wavelengths of 660 and 910 nm for red and infrared lights, respectively. The oximeter was connected to the acquisition apparatus (Power Lab, ADInstruments) linked to a computer software (LabChart 8, ADInstruments).

#### 2.3.4 Blood lactate concentration

Within 1 min after the RI task failure, capillary blood lactate concentrations ([Lac^−^]) were measured with a portable lactate analyzer (Lactate Scout, SensLab GmbH). A lancet was used to perform a pinprick after wiping the finger with an alcohol swab, and a 2 µl capillary sample of whole blood was collected and immediately analyzed.

### 2.4 Data analyses

#### 2.4.1 $\dot{V}$O_2_

$\dot{V}$O_2_ data were individually analyzed as previously described (Lamarra, Whipp, Ward, & Wasserman, 2017). Briefly, aberrant data points that were three standard deviations (*SD*) from the local mean were removed. Data were then linearly interpolated to 1 s intervals. $\dot{V}$O_2max_ was defined as the highest $\dot{V}$O_2_ computed from a 20‐s rolling average. Gas exchange threshold (GET) and RCP were identified by three independent investigators by examining raw respiratory data. Briefly, GET corresponded to the breakpoint in the $\dot{V}$O_2_‐to‐ $\dot{V}$CO_2_ relationship (i.e. V‐slope method) concomitant with an increase in the ventilatory equivalent of O_2_ and a leveling off of end‐tidal pressure of CO_2_ (PCO_2_) (Beaver, Wasserman, & Whipp, 1986). The RCP corresponded to the second disproportional increase (i.e. second breakpoint) in the V̇_E_/ $\dot{V}$O_2_ relation, where end‐tidal PCO_2_ began to fall after a period of isocapnic buffering (Whipp et al., 1989). In case of disagreement of more than 100 ml min^−1^, investigators would revaluate together the profiles until consensus was reached. To account for the circulatory transit time delay of deoxygenated hemoglobin from the active musculature to reach the lungs and the kinetics of $\dot{V}$O_2_, the MRT was calculated on an individual basis, as previously described (Iannetta, Murias, et al., 2019), using a customized function of a computer software (Origin, Origin Lab). A linear regression of the $\dot{V}$O_2_ versus PO relationship was fitted from the onset of the systemic rise in $\dot{V}$O_2_ until the previously established GET. The steady‐state $\dot{V}$O_2_ from the moderate step transition (i.e. 80 W for 6 min) was superimposed on the $\dot{V}$O_2_ versus PO relationship. Thereafter, the MRT corresponded to the difference between (a) the PO equivalent to the abscissa of the intersection between the $\dot{V}$O_2_ and the linear fit versus PO, and (b) the steady‐state $\dot{V}$O_2_ corresponding to 80 W (i.e. measured during the step transition) (Iannetta, Murias, et al., 2019).

#### 2.4.2 NIRS‐derived signals

**Figure 1:**
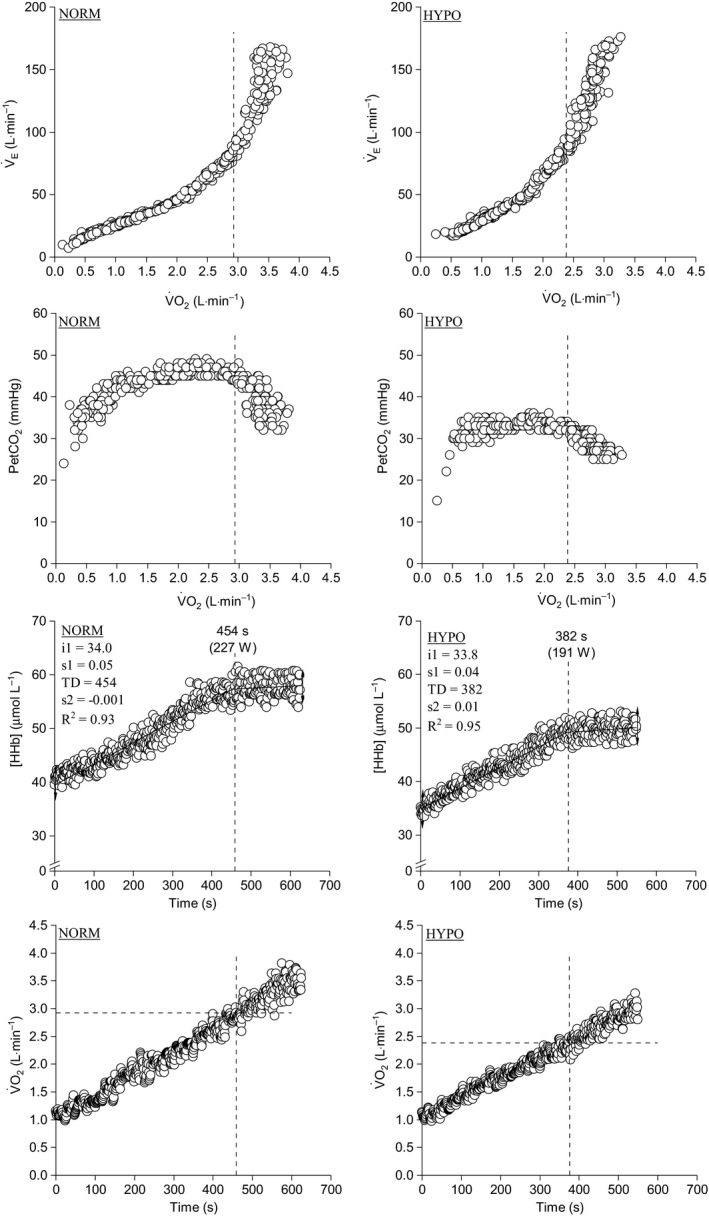
Identification of the RCP and the [HHb]_bp_ with associated $\dot{V}$O_2_ during the RI tests performed in normoxia and hypoxia. NORM, normoxia condition (FiO_2_ = 20.9%); HYPO, hypoxia condition (FiO_2_ = 16.0%); [HHb], deoxygenated hemoglobin; $\dot{V}$O_2_, oxygen uptake; V̇_E_, minute ventilation; PetCO_2_: end‐tidal partial pressure of CO_2_. Dashed vertical line indicates deoxygenated hemoglobin and respiratory compensation point occurrence among the different variables for the same participant in distinct FiO_2_ condition

The [HHb]‐time relationship for VL was modeled with the following piecewise equation that included two linear segments (e.g. “double linear”), as previously described (Spencer et al., 2012):

$$f=\text{if}\left(x<\text{TD},\;\;g\left(x\right),\;\;h\left(x\right)\right)$$

$$g\left(x\right)=i1+\left(s1\cdot x\right)$$

$$i2=i1+\left(s1\cdot \text{TD}\right)$$

h(x)=i2+[s2·(x-TD)]

fitftoy,

where *f* is the “double‐linear” function, *x* is time and *y* is [HHb], TD (i.e. time delay) is the time coordinate corresponding to the interception of the two regression lines (i.e. [HHb]_bp_), *i*1 and *i*2 are the intercepts of the first and second linear function, respectively and *s*1 and *s*2 are the slopes (i.e. SL1 and SL2, respectively). Model parameter estimates were determined by linear least‐square regression analysis in which the best fit was defined by minimization of the residual sum of squares (RSS) and highest coefficient of determination (*R* ^2^). The “double‐linear” fit was performed plotting the [HHb] data against PO with the fit starting at the onset of the systematic increase in the [HHb] signal until the last data point corresponding to the end of the RI test. Aberrant data that were 3 ± *SD* from the local mean were removed. The piecewise equation was selected to fit this specific range of data where the equation parameters (i.e. il, s1, s2, and TD) were not previously fixed and were automatically selected by the mathematical software. Thereafter, the $\dot{V}$O_2_ associated with the [HHb]_bp_ was identified after having “left‐shifted” the $\dot{V}$O_2_ data (Fontana et al., 2015). Finally, the end‐exercise SpO_2_ and StO_2_ (i.e. last 10% of the RI test) were utilized to compare the effectiveness of distinct FiO_2_ in altering the O_2_ saturation at the arterial and muscle level, respectively. Figure 1 shows a representative participant in whom the RCP and the [HHb]_bp_ were identified.

### 2.5 Statistical analyses

Descriptive data are presented as mean ± standard deviation (*SD*). Data normal distribution was tested by Shapiro–Wilk test and Q‐Q plots. End‐exercise PPO, $\dot{V}$O_2max_, SpO_2_, StO_2_, and [Lac^−^] were compared using paired samples *t* tests. The $\dot{V}$O_2_ and the PO associated with the RCP and the [HHb]_bp_ variables (i.e. absolute and relative values) and the [HHb] NIRS‐derived slopes before and after the [HHb]_bp_ (i.e. SL1 and SL2, respectively) were compared utilizing a two‐way repeated measures ANOVA. When *F* values were significant, Tukey *post hoc* were used to determine the loci of significant differences. The effect size was computed as partial eta squared ( $\eta_{p}^{2}$) for ANOVA comparisons [i.e. assuming the small (<0.02), medium (0.02–0.26) and large (>0.26) effect sizes] (Bakeman, 2005). For two group comparisons as Cohen D (*d*) [i.e. ranked as trivial (0–0.19), small (0.20–0.49), medium (0.50–0.79), and large (0.80 and greater) effect sizes] (Cumming, 2014). Bland–Altman plot analyses were used to compare the $\dot{V}$O_2_ and PO average errors and limits of agreement (LoA) between the RCP and [HHb]_bp_ at each FiO_2_ condition (Bland & Altman, 1986; Inglis et al., 2019). In addition, the association between values of $\dot{V}$O_2_ and PO at the RCP and the [HHb]_bp_ were tested by linear regression and Pearson's product moment correlation. Finally, the $\dot{V}$O_2_ and the PO associated with the RCP and the [HHb]_bp_ in HYPO condition were compared based on the delta change from NORM condition with a Pearson's product moment correlation. The significance level was set at *p* < .05. All statistical analyses were performed using a statistical software package (Statistica, version 10.0).

## 3 RESULTS

### 3.1 Ramp‐incremental physiological responses and [HHb] signal adjustment in NORM and HYPO conditions

$\dot{V}$O_2max_ was lower in HYPO (2.98 ± 0.36 L min^−1^) compared to NORM (3.39 ± 0.26 L min^−1^) (*p* < .001, *d* = −1.32). Similarly, PPO was lower in HYPO (282 ± 29 W) than in NORM (310 ± 19 W) (*p* = .002, *d* = −1.12). [Lac^−^] was not different between conditions (HYPO, 9.6 ± 2.6 mM L^−1^; NORM: 10.0 ± 2.3 mM L^−1^; *p* = .522, *d* = −0.16). SpO_2_ and StO_2_ in HYPO (81% ± 4% and 58% ± 11%, respectively) were lower than in NORM (90% ± 5% vs. 65% ± 10%, respectively) (*p* = .001 and 0.007, *d* = −1.85 and −0.63) at the last 10% of the RI test. The slope of the [HHb] NIRS signal before the [HHb]_bp_ (i.e. SL1; HYPO, 0.039 ± 0.022 µM s^−1^; NORM, 0.044 ± 0.024 µM s^−1^) was greater than after the [HHb]_bp_ (i.e. SL2; HYPO, 0.019 ± 0.022 µM s^−1^; NORM, 0.019 ± 0.020 µM s^−1^) (*p* = .003, $\eta_{p}^{2}$ = 0.583) but there was no difference between conditions (*p* = .562, $\eta_{p}^{2}$ = 0.034). The “double‐linear” fitting parameters for [HHb]_bp_ identification showed high values for goodness‐of‐fit within the data set, in accordance with a previous study (Spencer et al., 2012), as given by the RSS and *R* ^2^ for HYPO (RSS = 709 ± 269; *R* ^2^ = 0.92 ± 0.06) and NORM (RSS = 658 ± 247; *R* ^2^ = 0.93 ± 0.03) conditions.

### 3.2 The $\dot{V}$O_2_ and PO associated with the RCP and the [HHb]_bp_ variables between the different FiO_2_ conditions

The RCP and the [HHb]_bp_ associated with absolute and relative $\dot{V}$O_2_ and PO values are shown in Table 1. The absolute values for $\dot{V}$O_2_ associated with the RCP and the [HHb]_bp_ were lower in HYPO compared to NORM condition (*p* < .001, $\eta_{p}^{2}$ = 0.785) but there was no difference between variables within the same condition (*p* = .964, $\eta_{p}^{2}$ < 0.001). The % $\dot{V}$O_2max_ associated with the RCP and the [HHb]_bp_ were neither different between conditions (*p* = .235, $\eta_{p}^{2}$ = 0.069) nor variables within the same condition (*p* = .924, $\eta_{p}^{2}$ < 0.001). The absolute values for the PO associated with the RCP and the [HHb]_bp_ were lower in HYPO when compared to NORM (*p* < .001, $\eta_{p}^{2}$ = 0.826), but there was no difference between the RCP and the [HHb]_bp_ within the same condition (*p* = .848, $\eta_{p}^{2}$ = 0.001). The %PPO associated with RCP and [HHb]_bp_ in HYPO were lower compared to NORM (*p* < .001, $\eta_{p}^{2}$ = 0.575) but there was no difference between the RCP and the [HHb]_bp_ within the same condition (*p* = .756, $\eta_{p}^{2}$ = 0.004). The MRT obtained for HYPO (37 ± 10 s) and NORM (39 ± 13 s) conditions were not different (*p* = .363, *d* = −.329).

**Table 1:** Respiratory compensation point (RCP) and deoxyhemoglobin breakpoint ([HHb]_bp_) variables in each FiO_2_ condition

	NORM		HYPO	
	RCP	[HHb]_bp_	RCP	[HHb]_bp_
$\dot{V}$O_2_ (L·min^−1^)	2.75 ± 0.26* (2.24–3.26)	2.75 ± 0.28* (2.19–3.31)	2.35 ± 0.24 (1.89–2.81)	2.34 ± 0.26 (1.82–2.86)
$\dot{V}$O_2_ (%)	81 ± 5	81 ± 6	79 ± 6	78 ± 5
PO (W)	244 ± 29* (188–300)	241 ± 28* (186–294)	198 ± 37 (126–270)	197 ± 30 (138–255)
PO (%)	79 ± 6*	78 ± 7*	69 ± 7	69 ± 5

Note

Data are presented as mean ± *SD* and 95% confidence intervals in brackets.

Abbreviations: %, percentage of peak power output and $\dot{V}$O_2max_; [HHb]_bp_, deoxygenated hemoglobin breakpoint; HYPO, hypoxia condition (FiO_2_ = 16.0%); NORM, normoxia condition (FiO_2_ = 20.9%); $\dot{V}$O_2_, oxygen uptake; PO, power output; RCP, respiratory compensation point.

* Statistically different from HYPO (*p* < .05).

### 3.3 Correspondence between the RCP and the [HHb]_bp_ variables within the same FiO_2_ condition

**Figure 2:**
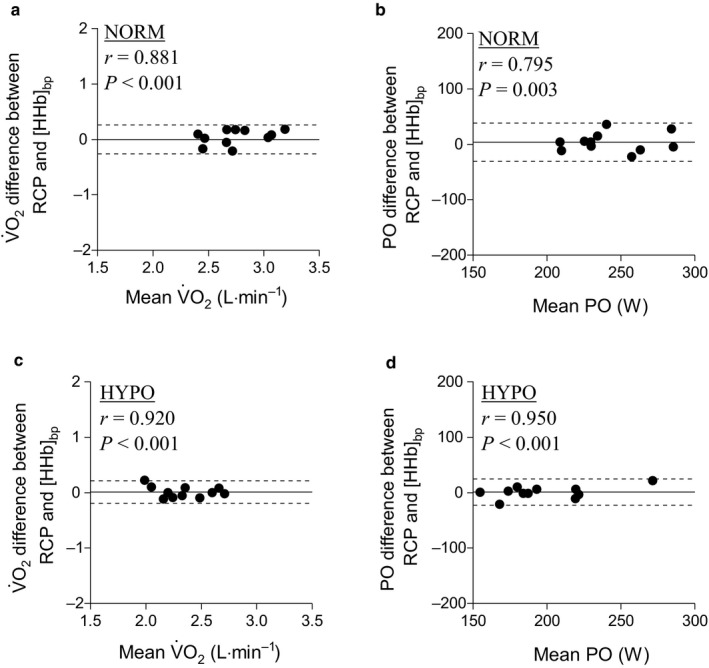
Bland–Altman plots comparison between the $\dot{V}$O_2_ and the PO associated with the respiratory compensation point (RCP) and the deoxyhemoglobin breakpoint ([HHb]_bp_) for each FiO_2_ condition. Black lines indicate mean difference error between the oxygen uptake ( $\dot{V}$O_2_) associated with RCP and [HHb]_bp_; Dashed black lines indicate lower and upper limits of agreement (±2 *SD*). (a) $\dot{V}$O_2_ associated with RCP and [HHb]_bp_ in normoxia condition (FiO_2_ = 20.9%); (b) Power output associated with RCP and [HHb]_bp_ in normoxia condition; (c) $\dot{V}$O_2_ associated with RCP and [HHb]_bp_ in hypoxia condition (FiO_2_ = 16.0%); (b) PO associated with RCP and [HHb]_bp_ in hypoxia condition

**Figure 3:**
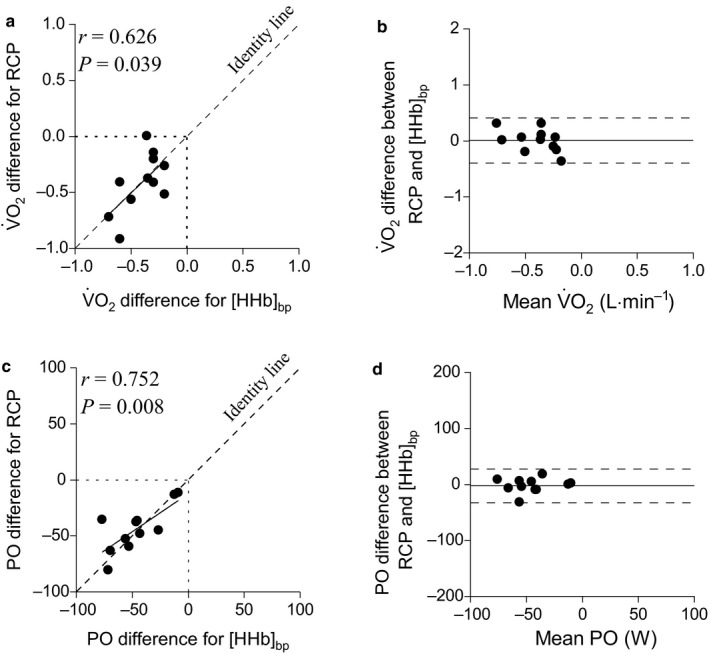
Correlation and Bland–Altman plots for the $\dot{V}$O_2_ and PO delta difference between hypoxia (HYPO) and normoxia (NORM) conditions for the respiratory compensation point (RCP) and deoxyhemoglobin breakpoint ([HHb]_bp_). (a) correlation for oxygen uptake ( $\dot{V}$O_2_) delta change for associated with the RCP and the [HHb]_bp_ between hypoxia and normoxia; (b) $\dot{V}$O_2_ delta change for the RCP and the [HHb]_bp_ between hypoxia and normoxia conditions; (c) correlation for power output (PO) delta change for associated with the RCP and the [HHb]_bp_ between hypoxia and normoxia; (d) PO delta change for the RCP and the [HHb]_bp_ between hypoxia and normoxia conditions. Black lines indicate mean difference error and dashed black lines indicate lower and upper limits of agreement

The comparison between the $\dot{V}$O_2_ and the PO associated with the RCP and the [HHb]_bp_ within the HYPO and NORM conditions are shown in the Bland–Altman plots (Figure 2). The $\dot{V}$O_2_ mean average error for HYPO (0.01 L min^−1^, LoA, lower: −0.19 L·min^−1^; upper, 0.21 L min^−1^; *p* = .761) (Figure 2a) and NORM (0.00 L min^−1^, LoA: lower, −0.26 L min^−1^; upper, 0.26 L min^−1^; *p* = .997) (Figure 2c) were not different from zero and strongly correlated. The PO mean average error bias for HYPO (1.1 W, LoA, lower: −23 W; upper, 25 W; *p* = .749) (Figure 2b) and NORM (3.6 W, LoA, lower: −31 W; upper, 38 W; *p* = .481) (Figure 2d) was also not different from zero and strongly correlated. The change in $\dot{V}$O_2_ and PO associated with the RCP and the [HHb]_bp_ in HYPO compared to NORM condition are shown in Figure 3. The $\dot{V}$O_2_ and PO associated with RCP and [HHb]_bp_ were equally decreased in HYPO compared to NORM, as demonstrated by a significant correlation for each variable (Figure 3a and c). Moreover, the mean average error bias for $\dot{V}$O_2_ (0.00 L min^−1^, LoA, lower: −0.39 L min^−1^; upper, 0.41 L min^−1^; *p* = .877) (Figure 3b) and PO (−2 W, LoA, lower: −32 W; upper, 27 W; *p* = .572) (Figure 3d) associated with the RCP and the [HHb]_bp_ in HYPO compared to NORM condition was not statistically different from zero.

## 4 DISCUSSION

This study investigated whether the strong relationship between the RCP and the [HHb]_bp_ that is typically observed in normoxia during a RI test (Fontana et al., 2015; Iannetta, Qahtani, Maturana, & Murias, 2017; Iannetta, Qahtani, Millet, et al., 2017; Inglis et al., 2019; Keir et al., 2015; Murias, Keir, Spencer, & Paterson, 2013) was affected by breathing hypoxic air (FiO_2_ 16%). The main and novel findings were that: (a) the RCP and [HHb]_bp_ were equally reduced during hypoxia in terms of both $\dot{V}$O_2_ and PO; (b) these changes were highly correlated, showing small and nonsignificant biases within each individual in each experimental condition (i.e. NORM and HYPO). Taken together, these results indicate that breathing a lower FiO_2_ during a RI test results in a proportional reduction in both the $\dot{V}$O_2_ and PO associated with the RCP and the [HHb]_bp_. These data further support the notion that these indices of intensity reflect a similar physiological response that is linked to metabolic changes within the active muscles.

In line with previous studies (Knight et al., 1992; Osawa et al., 2011; Richardson et al., 2006), the detrimental effects of hypoxia in the present study are evidenced by the reduced SpO_2_ and StO_2_, and lower $\dot{V}$O_2max_ and PPO throughout and at the end of RI test, respectively, compared to NORM. Most importantly, the earlier development of metabolic perturbations associated with exercise in hypoxia is evident in the attenuated $\dot{V}$O_2_ and PO at which RCP and [HHb]_bp_ occurred. Acute changes in FiO_2_ have detrimental effects on exercise performance and maximal aerobic capacity due to reduced O_2_ delivery and diffusive driving pressure of O_2_ at the muscular level (Knight et al., 1992; Osawa et al., 2011; Richardson et al., 2006). Thus, in order to maintain the adequate balance between O_2_ delivery and O_2_ utilization, several physiological adjustments need to occur (Calbet, Rådegran, Boushel, & Saltin, 2009). For example, for a given absolute submaximal exercise intensity, there is a greater cardiac output and increased local vasodilatory responses when compared to normoxia (Calbet et al., 2009). In fact, it has been suggested that the hypoxic‐induced hyperemia response is proportional to the hypoxia‐induced fall in arterial O_2_ content (Casey & Joyner, 2011). However, as the exercise intensity increases, these adjustments cannot counteract the reduced driving pressure of O_2_ at the capillary‐to‐muscle interface, limiting mitochondrial ATP resynthesis rate (Richardson, Leigh, Wagner, & Noyszewski, 1999). Thus, during a RI test to exhaustion in hypoxic conditions, there is an earlier reliance on anaerobic resources to sustain the ATP requirement when compared to normoxia (Connett, Honig, Gayeski, & Brooks, 1990; Linnarsson, Karlsson, Fagraeus, & Saltin, 1974). This is also due to increased circulation of catecholamines (i.e. epinephrine and noradrenaline) and an earlier recruitment of type II fibers which increase the glycolytic contribution to the ATP resynthesis in hypoxia compared to normoxia (Moritani, Sherman, Shibata, Matsumoto, & Shinohara, 1992; Osawa et al., 2011). This is evidenced by previous studies showing greater lactate efflux from the muscle to blood and greater depletion of intramuscular phosphagen resources at a given submaximal absolute work rate in hypoxia compared to normoxia (Hogan, Richardson, & Haseler, 1999; Linnarsson et al., 1974). Additionally, the present results and previous findings (Azevedo et al., 2020; Osawa et al., 2011) have consistently shown that the occurrence of [HHb]_bp_ is associated with lower absolute PO but the rate of change on [HHb] signal (i.e. SL1 and SL2) is not different either before or after the [HHb]_bp_ (Azevedo et al., 2020). Thus, based on those abovementioned mechanisms, it would be plausible to suggest that breathing hypoxic air promotes a “left‐shift” in the physiological responses throughout a RI test when compared to normoxia.

In relation to the NORM condition, the present results are in line with previous studies which have shown a correspondence between the RCP and the [HHb]_bp_ both in terms of $\dot{V}$O_2_ and PO in normoxia condition (Fontana et al., 2015; Iannetta, Qahtani, Millet, et al., 2017; Keir et al., 2015). Murias et al. (2013) were the first to demonstrate that the RCP and the [HHb]_bp_ occurred at the same $\dot{V}$O_2_ throughout a RI test, which was later confirmed by several other studies in large and heterogenous samples of individuals and in a test–retest conditions (Fontana et al., 2015; Iannetta, Passfield, et al., 2018; Iannetta, Qahtani, Millet, et al., 2017; Inglis et al., 2019; Keir et al., 2015). In direct connection with the goal of this investigation, the present data further confirm the correspondence between the RCP and [HHb]_bp_ when exercising under distinct FiO_2_ conditions. Based on the Bland–Altman plots analysis, the bias obtained for the $\dot{V}$O_2_ associated with the RCP and [HHb]_bp_ was not different from zero neither in NORM (0.00 L min^−1^) nor in HYPO conditions (0.01 L min^−1^) (Figure 2). These biases are similar to those previously reported in other studies (Fontana et al., 2015; Iannetta, Qahtani, Maturana, et al., 2017). Similarly, the PO associated with the RCP and [HHb]_bp_, displayed biases that were small in NORM (i.e. 3.6 W) and HYPO (i.e. 1.1 W), and similar to previously reported findings (Inglis et al., 2019). In addition to this, the $\dot{V}$O_2_ and the PO change in HYPO from NORM condition for RCP and [HHb]_bp_ were significantly correlated (Figure 3a and c), showing a bias that was not different from zero (Figure 3b and d). This is an important finding, as it indicates that both, the $\dot{V}$O_2_ and the PO associated with the RCP and the [HHb]_bp_, were negatively affected by the same degree in hypoxia.

The fact that the RCP and [HHb]_bp_ continued to occur at the same metabolic rate, independently of the FiO_2_, provides further support to the idea that these events might be triggered by common physiological responses occurring at the systemic and muscular level (Fontana et al., 2015; Keir et al., 2015). In other words, both the RCP and the [HHb]_bp_ are linked to a metabolic rate corresponding to the transition from the heavy to the severe exercise intensity domain (Keir et al., 2015), which elicits important metabolic changes as a consequence of metabolites accumulation within the muscle and bloodstream (Whipp et al., 1989). For example, up to a critical metabolic rate, increases in ventilation are sufficient to constraint the increases in arterial [H^+^] linked to CO_2_ production (Wasserman, Beaver, Sun, & Stringer, 2011). However, the increase in rate of [H^+^] accumulation, when progressively increasing the intensities above the critical metabolic rate, exceeds the capacity of this buffering system, which results in a more rapid accumulation of [H^+^], triggering a reflex increase in ventilation (i.e. the RCP) as a result of its uncoupling in relation to V̇CO_2_. Even though the exact mechanisms underpinning the hyperventilatory response throughout a RI test, and more specifically above the RCP, are still under debate (Nicolò, Marcora, & Sacchetti, 2020), there are several relevant systemic and local muscular changes that may play a direct effect (Wasserman et al., 2011; Whipp et al., 1989). Furthermore, the transition into the severe intensity domain is characterized not only by an increase in [H^+^], with a subsequent reduction in pH, but also by a decrease in the partial pressure of O_2_ within the active muscles, which are known to favor local vasodilation (Casey & Joyner, 2011) and, consequently, redistribution of blood flow to the active tissues. Accordingly, it has been suggested that increased blood flow distribution to the vastus lateralis muscle might be the main mechanism responsible for the observed plateau in the [HHb] signal during ramp‐incremental exercise (Azevedo et al., 2020; Iannetta, Okushima, et al., 2018; Inglis et al., 2017; Murias, Spencer, et al., 2013), as the improved local blood flow would reduce the need to further increase O_2_ extraction to support the continued increase in muscle $\dot{V}$O_2_. This is in line with findings from animal models, whereby the greater activation of type II fibers when surpassing the heavy‐to‐severe boundary of exercise intensity triggers disproportionate increases in blood flow in these fibers (Copp, Hirai, Musch, & Poole, 2010). However, although in humans the superficial portions of the vastus lateralis (which are the target of the NIRS light) are characterized by a greater proportion of type II muscle fibers (Johnson, Polgar, Weightman, & Appleton, 1973), this interpretation must be taken with caution because, since the human skeletal muscle is characterized by a mosaic of different fiber types, which may present disparate vascular dynamics and not necessary demonstrate such abrupt changes. In this regard, the leveling off of the [HHb] signal in the vastus lateralis muscle has been demonstrated not to be depth dependent (Iannetta, Okushima, et al., 2018) or affected by differential muscle recruitment patterns (Okushima et al., 2020) and exercise modes (Iannetta, Passfield, Qahtani, MacInnis, & Murias, 2019), which would suggest that this phenomenon may not be ascribed to a unique fiber type population.

Alternatively, it has been proposed that even though the RCP and the [HHb]_bp_ might share somewhat similar underpinning mechanisms, they manifest an order of occurrence (Boone, Barstow, et al., 2016; Boone, Vandekerckhove, Coomans, Prieur, & Bourgois, 2016). Accordingly, as intensity increases there is an increasing recruitment of type II fibers leading to the development of metabolic acidosis that triggers the hyperventilatory response (i.e. RCP) and, subsequently, the occurrence of the [HHb]_bp_ as a consequence of the attainment of a supposedly maximal O_2_ extraction (Boone, Barstow, et al., 2016; Boone, Vandekerckhove, et al., 2016). It should be noted, however, that one of these studies has actually shown that the RCP and the [HHb]_bp_ occurred at the same metabolic rate (Boone, Barstow, et al., 2016). Furthermore, it has been recently discussed that discrepancies in views as to whether or not the RCP and the [HHb]_bp_ are expressed simultaneously or in sequential order might be explained by the approach used to account for the MRT, as detailed elsewhere (Boone, Caen, Vermeire, Bourgois, & Bourgois, 2019; Keir et al., 2019).

### 4.1 Experimental considerations

It should be noted that the goal of this study was to determine whether changes (or the lack thereof) in the $\dot{V}$O_2_ and PO associated with the RCP and the [HHb]_bp_ when breathing hypoxic air were proportional. In this context, it could be argued that including a hyperoxia condition would have provided additional insights. In fact, this study originally included such condition and, when the RCP and the [HHb]_bp_ in hyperoxia were analyzed (data presented elsewhere (Azevedo et al., 2020)), they occurred at the same % $\dot{V}$O_2max_ and %PPO compared to normoxia condition. However, as stated in our previous manuscript (Azevedo et al., 2020) and also reported by other studies (Amann et al., 2007; Oussaidene et al., 2013; Rausch, Whipp, Wasserman, & Huszczuk, 1991; Ulrich et al., 2017), measurements of $\dot{V}$O_2_ using current systems are often not valid during hyperoxic conditions, (even after attempting corrections as proposed elsewhere (Lang, Herold, Kraft, Harth, & Preisser, 2018)). Therefore, the inclusion of the hyperoxia condition is not appropriate since the $\dot{V}$O_2_ data are not physiologically justifiable.

In conclusion, the present study demonstrated that the RCP and the [HHb]_bp_ during a RI test occur at the same metabolic rate and PO, even under distinct FiO_2_ conditions that alter the absolute values at which these intensity indices occur during the test. Furthermore, and most importantly, the magnitudes of the changes in both the RCP and the [HHb]_bp_ from normoxic to hypoxic conditions were proportional on an individual basis. Taken together, data from the present study reinforce the idea that the RCP and the [HHb]_bp_ parameters represent a similar physiological response and that the mechanisms underpinning their occurrence are likely linked to metabolic changes that take place when surpassing the heavy‐to‐severe boundary of exercise intensity.

## 5 PERSPECTIVES AND SIGNIFICANCE

This study is the first to evaluate the correspondence between the RCP and the [HHb]_bp_ during a RI test under distinct FiO_2_ conditions (i.e. normoxia and hypoxia). It was found that the $\dot{V}$O_2_ and PO associated with the RCP and the [HHb]_bp_ were not different within conditions but both reduced under hypoxia condition. Most importantly, the changes in hypoxia, in terms of the $\dot{V}$O_2_ and PO, negatively affected the RCP and the [HHb]_bp_ in a similar magnitude compared to normoxia. Thus, the present data reinforce the idea that both markers of intensity represent a similar physiological response that delimitates the metabolic rate associated with the transition from the heavy to the severe intensity domain. These findings have important implications to further our understanding on the physiological underpinnings of these markers.

## CONFLICT OF INTEREST

The authors declare no conflict of interest.
